# SMARCAL1: a new target for taming tumor immune evasion

**DOI:** 10.1002/mco2.730

**Published:** 2024-09-07

**Authors:** Ting Xiao, Shiqi Li, Xinghua Long

**Affiliations:** ^1^ Department of Laboratory Medicine Zhongnan Hospital of Wuhan University Wuhan China

1

The viability and development of cancer cells depend on their ability to effectively evade the surveillance of the immune system. To achieve this, cancer cells alter the expression or function of specific molecules to become “invisible”. In a recent elegant study in *Cell*, Leuzzi et al.^1^ unveiled a novel player in the intricate game of tumor immune evasion: SMARCAL1, linked to DNA repair, emerges as a dual regulator with a surprising ability to modulate both innate immune signaling and PD‐L1 expression. This work not only sheds light on the complex interplay between DNA damage response, innate immunity, and immune checkpoint regulation, but also identifies a potential new target for cancer immunotherapy.

Using a CRISPR–Cas9 screen, the authors identified SMARCAL1 as one of the sgRNA‐targeted genes that are distinctly enriched in IRF3^High^/PD‐L1^Low^ cells (Figure 1A). Interferon regulatory factor 3 (IRF3) is a central signaling mediator of the innate immune signaling pathway, selected as a screening factor. In the innate immunity cGAS–STING pathway, abnormally exposed double‐stranded DNA (dsDNA) in the cytoplasm binds to cGAS to produce cGAMP.^2^ cGAMP binding by STING is activated, recruiting TANK‐binding kinase to phosphorylate IRF3 leading to its nuclear translocation and the induction of type I interferons (IFNs), which then triggers the expression of interferon stimulated genes (ISGs) expression to enhance cellular autonomous defense mechanisms.^3^ Furthermore, PD‐L1 can undergo upregulation in antigen‐presenting cells following IFN‐γ stimulation, which leads to immune tolerance. This observation provides fresh perspectives on the significant role of SMARCAL1 in modulating IRF3‐mediated immune stimulation and the expression levels of the immune checkpoint PD‐L1.

**FIGURE 1:**
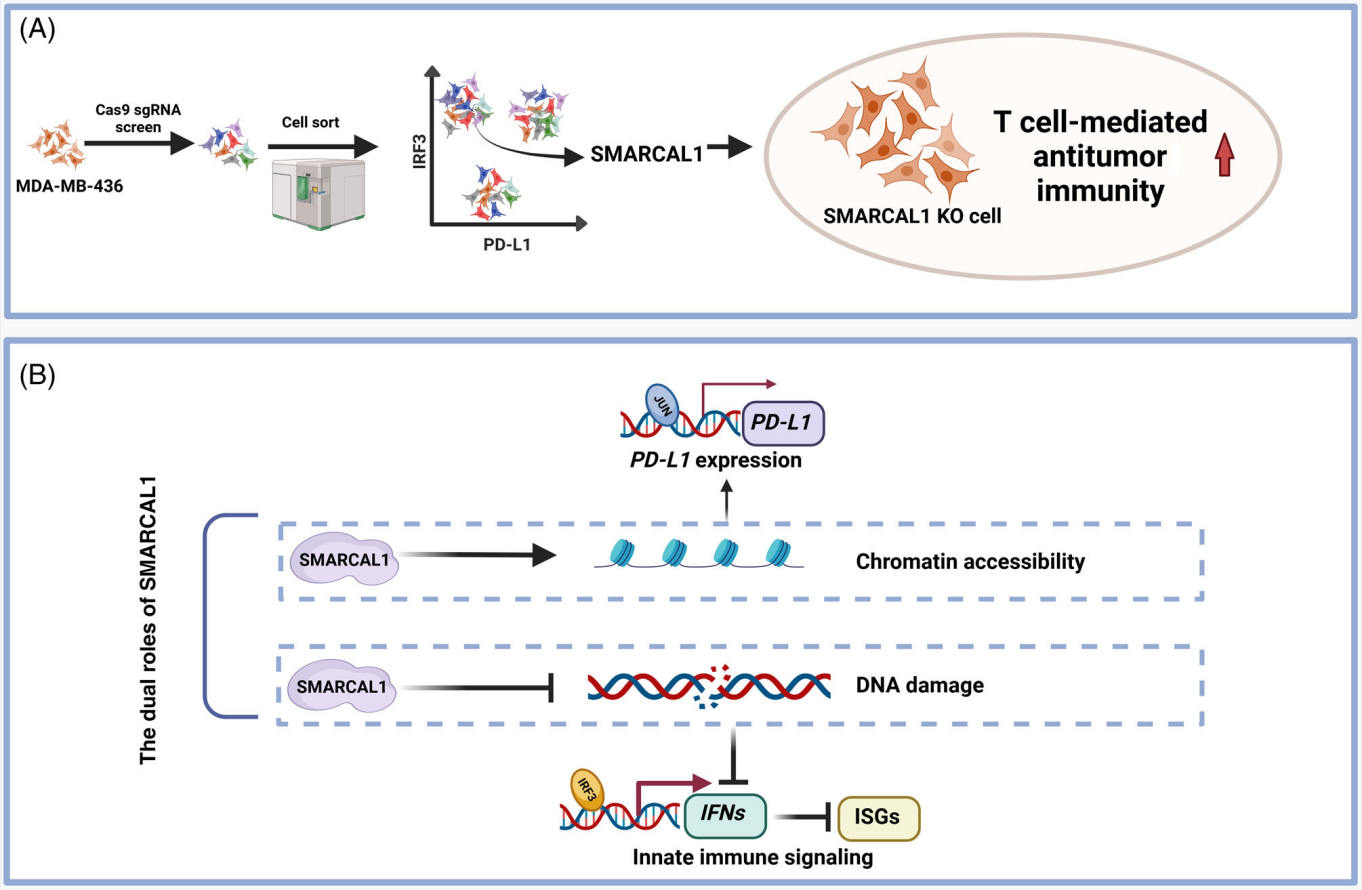
(A) SMARCAL1, identified via CRISPR–Cas9, is enriched in cell subgroups with high nuclear IRF3 and low PD‐L1 levels, and SMARCAL1‐KO cells are more susceptible to T‐cell‐mediated killing. (B) SMARCAL1 operates through two mechanisms: partnering with JUN to enhance chromatin accessibility and PD‐L1 expression, and maintaining genomic stability to reduce innate immune stimulation from abnormal cytoplasmic DNA (created with BioRender.com [https://biorender.com]).

SMARCAL1 is an ATP‐dependent, SWI/SNF‐related annealing helicase that stabilizes replication forks during DNA damage. Mutations in this gene are responsible for Schimke immune‐osseous dysplasia, an autosomal recessive disorder characterized by T‐cell immunodeficiency and growth dysfunctions.^4^ In this study, SMARCAL1 operates on dual fronts. First, it dampens the cGAS–STING pathway, which is triggered by abnormal DNA in the cytoplasm. Chromosomal instability is a primary source of cytosolic dsDNA, a hallmark of cancer cells.^2^ Second, it promotes PD‐L1 expression, which shields tumors from T cell attack and allows cancer cells to enhance their viability and metastatic potential. SMARCAL1‐deficient tumor cells show higher levels of IRF3 phosphorylation and reduced levels of PD‐L1. This is consistent with the screening results, indicating that SMARCAL1 deficiency simultaneously induces IRF3 activation to promote the cGAS–STING pathway and downregulates PD‐L1. This dual functionality renders SMARCAL1 an exceptional and potentially powerful candidate for immunotherapy.

Mechanistically, SMARCAL1 can facilitate the repair of collapsed DNA replication forks, alleviating the DNA replication stress caused by oncogenes.^5^ Loss of SMARCAL1 induces genome instability and leads to micronuclei formation. Disruption of the micronuclear envelopes exposes dsDNA to the cytosol, leading to the activation of the cGAS–STING signaling pathway. Furthermore, SMARCAL1 does not directly mediate chromatin remodeling. Notably, SMARCAL1 upholds chromatin accessibility of P3 element located downstream of the PD‐L1 transcription start site through its ATPase activity, and cooperates with the AP‐1 family member JUN to efficiently promote PD‐L1 expression. The research opens a new window to reveal how a single molecule plays a dual role in cancer cells, both hiding itself from the immune system's attack and facilitating the immune system's fight back under certain conditions. This discovery not only provides new insights into the complex mechanisms of cancer, but also opens up the possibility of developing more effective cancer treatment strategies.

In mouse models, SMARCAL1 deficiency significantly inhibited tumor growth and increased animal survival. Notably, SMARCAL1 is cGAS‐dependent for antitumor immunity and enhances T cell‐mediated antitumor competence. Due to the lack of accessible P3 regions in B16/F10 mouse cells, SMARCAL1‐deficiency‐mediated regulation of PD‐L1 levels could not be assessed for antitumor effects in the study. Interestingly, PD‐L1 downregulation induced by SMARCAL1 deletion in MDA‐MB‐436 cells made them more sensitive to CD8^+^ T cell‐mediated killing, which could be partially rescued by PD‐L1 overexpression. Moreover, treatment of SMARCAL1‐deficient B16/F10 tumors with anti‐PD‐L1 antibody or anti‐CTLA‐4 antibody significantly reduced tumor growth and improved animal survival. Combined treatment with both antibodies enhanced the effect. SMARCAL1 deficiency has a synergistic effect with immune checkpoint blockade treatment for antitumor immunity.

Immune checkpoint inhibitors (ICIs) are the mainstay of tumor immunotherapy, blocking cancer cell evasion mechanisms to enhance the immune system's attack on cancer cells. Nevertheless, inherent or acquired tumor resistance to ICIs and treatment‐related toxicity limit their clinical application.^6^ The dual mechanism of action of SMARCAL1 may be one of the key factors in enhancing the efficacy of ICIs. Targeting SMARCAL1 can synergize with ICIs to treat tumors, providing a novel and effective approach to tumor immunotherapy. Active DNA‐dependent ATPase A domain inhibitor (ADAADi) targets the ATPase domain of SMARCAL1, which was identified as a potent inhibitor of ADAAD, the bovine homologue of SMARCAL1, but SMARCAL1 does not determine the cellular response to ADAADi.^7^ Although there was no significant correlation between SMARCAL1 expression and cellular responsiveness to ADAADi, ADAADi provides a viable way to target inhibition of SMARCAL1 for tumor treatment.

Although intriguing, several questions persist. Initially, SMARCAL1‐mediated regulation of PD‐L1 in cancer patients could not be fully mimicked due to the study's heavy reliance on preclinical models, and the fact that SMARCAL1 does not affect the levels of PD‐L1 in mouse cells. Further evaluation of SMARCAL1‐mediated antitumor effects in mouse tumor models is needed. And it is required to conduct comprehensive research across various types of cancers. Second, minimizing off‐target effects and ensuring safety using known and newly developed SMARCAL1 inhibitors is a demanding task. Finally, further exploration is needed to achieve synergistic treatments combining SMARCAL1 targeting with other immunotherapies to enhance clinical benefits.

Overall, the work of Leuzzi et al.^1^ sheds light on a previously unknown mechanism of tumor immune evasion and highlights the potential of targeting SMARCAL1 as a novel therapeutic strategy (Figure 1B). Targeted inhibition of SMARCAL1 induces cancer innate immune signaling while downregulating PD‐L1 levels, resulting in a double whammy for cancer and better cancer treatment outcomes. Further investigation is required to elucidate the precise mechanisms through which SMARCAL1 controls PD‐L1 expression and its interactions with other gene targets. Additionally, exploring the potential of SMARCAL1 inhibitors or SMARCAL1‐targeting therapies in combination with existing immunotherapies is crucial. This approach is important for translating these findings into clinical applications and propelling them toward the advancement of efficacious cancer therapeutics.

## AUTHOR CONTRIBUTIONS

**Ting Xiao**: drafted the manuscript. **Shiqi Li**: drew the figure. and **Xinghua Long**: drafted and reviewed the manuscript. All the authors read and approved the final manuscript.

## CONFLICT OF INTEREST STATEMENT

The authors declare that they have no conflict of interest.

## ETHICS STATEMENT

Not applicable.

## Data Availability

Not applicable.
